# Reply to van Haastert: Local competition between Ras/actin-driven protrusions

**DOI:** 10.1073/pnas.2601215123

**Published:** 2026-03-06

**Authors:** Albert Alonso, Julius B. Kirkegaard, Robert G. Endres

**Affiliations:** ^a^Niels Bohr Institute, University of Copenhagen, Copenhagen 2100, Denmark; ^b^Department of Computer Science, University of Copenhagen, Copenhagen 2100, Denmark; ^c^Department of Life Sciences and Centre for Integrative Systems Biology and Bioinformatics, Imperial College London, London SW7 2AZ, United Kingdom

We thank van Haastert for his thoughtful Letter regarding our recent PNAS paper on pseudopod-based chemotaxis strategies in shallow gradients (1). Our work is a conceptual model of how local, competing protrusive “options” can implement robust directional decision-making in noisy shallow gradients (2). The core ingredient is competition for a shared, effectively limited resource—implemented in our model as “actin,” intended generically to include actin monomers and upstream regulators that control polymerization and stabilization.

Van Haastert raises two main concerns: i) that “true” pseudopod splitting is rare, and ii) that actin is not limiting because mutants with multiple pseudopods exhibit similar protrusion size and dynamics. We believe both points can be reconciled with our framework.

First, the apparent disagreement on splitting frequency largely reflects definitions and denominators. Andrew and Insall emphasize biased selection between concurrently extending protrusions during chemotaxis (3), whereas Bosgraaf and van Haastert focus on the global statistics of pseudopod initiation and classify only a subset of bifurcations as “Y-splits” (two simultaneously extending fronts) (4). Importantly, van Haastert’s own counts indicate that “rare” Y-splits comprise ~9 to 10% of pseudopod events but that cells spend ~23 to 25% of protrusive time with two extending fronts. Thus, even if Y-splits are a minority by event count, competitive two-front states may occupy a substantial fraction of the dynamics and could plausibly be enriched in decision-making moments—consistent with selection-based chemotaxis in shallow gradients. [This is also in line with analysis of microfluidic experiments by one of us, in which pseudopod splitting emerges as a prominent, though not dominant, cell morphology (5)]. The more common single-front pseudopods may promote migration between decision-making events or that actual cells use a mixed strategy.

Second, while we presented our model through a “limited actin pool,” the model is agnostic to what is exhausted. It represents any shared capacity that couples competing protrusions—such as availability of active regulators, branching machinery, membrane or tension constraints, or localized signaling components. In this sense, van Haastert’s Ras/actin excitable patch framework is not in conflict with our model: both describe a local competition in which one option is stabilized and others are suppressed. Whether one labels the competing entities as “pseudopods” or “Ras/actin patches that later form pseudopods” is a matter of mechanistic placement along the pathway, not of principle.

Finally, we agree with van Haastert that local receptor input can bias the growth of a Ras/actin patch before visible protrusion. Our work addresses the complementary question of how competition between locally amplified options can implement robust, low-information decision-making strategies and explains summary behavioral statistics (including response scaling) without requiring a globally coordinated chemical compass.

Van Haastert explains that “In the rare cases that cells extend multiple pseudopods, retraction of the pseudopod not facing the gradient may contribute to orientation in shallow gradients.” That sentence echoes the core mechanism of our model, focusing on those decision-making moments.

We therefore view the Letter as refining the biological interpretation (Ras/actin patches upstream of protrusions) of our simple model for competitive decision-making in shallow gradients.

## References

[1] P. J. M. Van Haastert, Local Ras/actin signaling orients individual pseudopods in shallow gradients. Proc. Natl. Acad. Sci. U.S.A. **123**, e2526852123 (2026).41790948 10.1073/pnas.2526852123

[2] A. Alonso, J. B. Kirkegaard, R. G. Endres, Persistent pseudopod splitting is an effective chemotaxis strategy in shallow gradients. Proc. Natl. Acad. Sci. U.S.A. **122**, e2502368122 (2025). 40339116 10.1073/pnas.2502368122 PMC12088397

[3] N. Andrew, R. H. Insall, Chemotaxis in shallow gradients is mediated independently of PtdIns 3-kinase by biased choices between random protrusions. Nat. Cell Biol. **9**, 193–200 (2007). 17220879 10.1038/ncb1536

[4] L. Bosgraaf, P. J. M. Van Haastert, The ordered extension of pseudopodia by amoeboid cells in the absence of external cues. PLoS One **4**, e5253 (2009). 19384419 10.1371/journal.pone.0005253 PMC2668753

[5] L. Tweedy, B. Meier, J. Stephan, D. Heinrich, R. G. Endres, Distinct cell shapes determine accurate chemotaxis. Sci. Rep. **3**, 2606 (2013). 24008441 10.1038/srep02606 PMC3764443

